# Dental care and services of children and young people with cerebral palsy in Australia: A comprehensive survey of oral health‐related quality of life

**DOI:** 10.1111/scd.13098

**Published:** 2025-01-04

**Authors:** Karen Lansdown, Kim Bulkeley, Margaret McGrath, Michelle Irving, Claudia Zagreanu, Hayley Smithers‐Sheedy

**Affiliations:** ^1^ Department of Oral Health, School of Clinical Sciences, Faculty of Health and Environmental Sciences Auckland University of Technology Auckland New Zealand; ^2^ School of Dentistry, Faculty of Medicine and Health The University of Sydney Sydney Australia; ^3^ Sydney School of Health Sciences, Faculty of Medicine and Health The University of Sydney Sydney Australia; ^4^ School of Clinical Therapies, College of Medicine and Health University College Cork Cork Ireland; ^5^ Office for Health and Medical Research NSW Ministry of Health Sydney Australia; ^6^ Cerebral Palsy Alliance Research Institute, Specialty of Child & Adolescent Health, Sydney Medical School, Faculty of Medicine & Health The University of Sydney Sydney Australia

**Keywords:** cerebral palsy, COHIP‐SF 19, dental care and services, OHRQoL

## Abstract

**Aims:**

To investigate caregiver‐reported dental care experiences and oral health‐related quality of life (OHRQoL) of children and young people with cerebral palsy (CP).

**Methods:**

Between May and August 2023, caregivers of children and young people from three Australian states were invited to complete questionnaires, including the Child Oral Health Impact Profile (COHIP‐SF 19).

**Results:**

Sixty‐eight caregivers participated in the survey. Most children and young people had spastic CP (69.1%) with unilateral spasticity most common (51.1%). The COHIP‐SF 19 average score was 51.9% ± 12.4, indicating moderate OHRQoL. Gender, communication, oral health daily routine, participation in dental exams and treatment, special arrangements needed to attend the practice, and urgent dental care due to pain or other problem(s) all significantly impacted OHRQoL (*p* < 0.05).

**Conclusion:**

OHRQoL of children and young people with CP is moderately impacted, as indicated by the COHIP‐SF 19 scores. To improve OHRQoL in this population group, it is crucial to prioritize key factors such as upskilling dental professionals and creating more inclusive dental environments.

## 1 INTRODUCTION

Cerebral palsy (CP) describes a group of movement disorders resulting from damage or injury to the developing brain. While these disorders are permanent life‐long conditions, they are not unchanging.^1^ People with CP can also experience associated conditions including but not limited to intellectual impairment and epilepsy.^2^ The birth prevalence of CP in high‐income countries is estimated to be 1.6 per 1000 live births, with a noticeably higher estimate of 3.4 per 1000 live births reported in low‐ and middle‐income countries.^3^

Neuromuscular issues stemming from CP can lead to alterations in orofacial structures, as well as difficulties with swallowing (dysphagia) and excessive drooling (sialorrhea).^4^ These neuromuscular impairments along with motor limitations, can compromise the ability of individuals with CP to maintain oral hygiene. Individuals may struggle to brush their teeth effectively, which can contribute to oral health problems such as dental caries, periodontal disease, and other oral health concerns.^5^, ^6^, ^7^, ^8^, ^9^, ^10^ Oral health issues can impact social interactions, self‐esteem, and overall well‐being, contributing to a diminished quality of life.^11^

Emerging evidence from research conducted in low‐ and middle‐income countries, suggests that people with CP may have poorer oral health‐related quality of life (OHRQoL) compared to their peers.^12^, ^13^, ^14^ It is not known if this is the case in high‐income countries as no studies to date have explored OHRQoL for children and young people with CP.^5^

The 2023 Senate Select Committee report highlighted significant barriers for people with disabilities in Australia, including a shortage of accessible dental services.^15^ The report found that only 26 specialists treat patients with disabilities nationwide, with some regions having no specialists.^15^ However, despite these concerns, research regarding access to dental care for people with CP, is limited with a recent scoping review finding a lack of evidence about access challenges for people with CP.^5^ No studies have yet addressed dental services for children and young people with CP in Australia, and currently, there is limited knowledge about their access to these services.^5^, ^15^

This study aimed to investigate (1) the OHRQoL and (2) the experiences of Australian children and young people with CP accessing dental care and services, as reported by their caregivers.

## 2 METHODS

This cross‐sectional study used purposive sampling to investigate the OHRQoL of children and young people in Australia with CP. The research team collaborated with three advocates with lived experience of CP to inform the study's development and reporting. Ethical approval was obtained from The University of Sydney [2023/127] and CP Alliance governance approvals [2023_04_01]; [CPL‐2023‐001].

### 2.1 Participants

Inclusion criteria required caregivers who were proficient in English, had functional vision and access to a computer or device for completing the questionnaire, and could consent to report on behalf of their children and young people aged 7–17 years of age with CP. Participants who were registered and had consented to receive research invitations were recruited from CP registers in New South Wales/Australian Capital Territory, Queensland, and Victoria. Invitations were emailed to approximately 2200 caregivers and promoted through newsletters and emails from May to August 2023, with reminder e‐mails sent after 8 weeks by two registers. Participation was voluntary, ensuring confidentiality and anonymity throughout the process. To prevent duplicate entries, participants provided their initials (First, Middle, and Last) along with their postcode. Based on recruitment information from approximately 2000 people, a multi‐regression analysis with an effect size of 0.20, a statistical power level of 0.8, and a probability level of 0.05 was utilized. The plan was to recruit approximately 70 participants to the study.

### 2.2 Data collection

Both informed consent and participant survey data were collected and managed using Research Electronic Data Capture (REDCap), a secure, web‐based platform hosted at The University of Sydney. REDCap is specifically designed to support data capture for research studies while ensuring compliance with data security and privacy requirements.^16^, ^17^

#### 2.2.1 Demographic and clinical information

The survey included three sections, the first section included 13 questions on participants' demographics and clinical details. Country of birth was categorized as Australia or overseas. Rural and remote postcodes were categorized using the Australian Statistical Geography Standard (Major Cities, Inner Regional, Outer Regional Australia, Remote/Very Remote)^18^ ^,^ and socio‐economic status was recorded using the Socio‐Economic Indexes for Areas (SEIFA)^19^ using quintiles, with one being the most disadvantaged and five the most advantaged. Information on CP, including motor type (Spastic, Dyskinetic, Ataxic) and spastic topography (unilateral or bilateral) was recorded. Functional mobility was categorized using the Gross Motor Function Classification System (GMFCS) collapsed into “Ambulant” (levels I‐II) and “Supported mobility” (levels III‐V).^20^ Manual ability was categorized using the Manual Ability Classification System (MACS) and collapsed into two categories I‐II and III‐V.^21^ Speech intelligibility was assessed with the Viking Speech Scale (VSS) and collapsed into two categories I‐II and III‐IV.^22^ Functional communication was classified using the Communication Function Classification System (CFCS) collapsed into two levels I‐II and III‐V.^23^ Comorbidities, such as hearing impairments and epilepsy, including anti‐seizure medication use, were also recorded.

#### 2.2.2 Child oral health questionnaire

The validated Child Oral Health Impact Profile (COHIP‐SF 19) was used to evaluate OHRQoL in children and young people with CP via proxy report.^24^ The survey includes 19 questions, across three domains, oral health well‐being, functional well‐being, and social‐emotional well‐being. The Oral Health Well‐being subscale consists of five items, with scores ranging from 1 to 16, assessing the impacts of oral health on functional aspects of life, such as pain, discolored teeth, bleeding gums, etc. The Functional Well‐being subscale consists of four items evaluating daily activities such as difficulty eating foods, sleeping, keeping teeth clean, etc. The Social Emotional Well‐being subscale includes 10 items, with scores ranging from 8 to 40, assessing emotional and social impacts, for example avoiding smiling, looking different, and being teased or bullied. Two positively worded questions were rated on a Likert scale from 0 (“never”) to 4 (“almost all the time”), and 17 negatively worded questions were reversed on the same scale. Higher scores on the Likert scale indicated a more positive OHRQoL.^25^

#### 2.2.3 Experiences of dental care and services

In the third section, data on dental care experiences for participants with CP were collected using Liu et al.’s questionnaire, with “disability” replaced by “CP”.^26^

### 2.3 Statistical analysis

All data were analyzed with SPSS Version 29.^27^ Internal consistency of the COHIP‐SF 19 was measured with Cronbach's alpha, requiring a minimum value of 0.7 for satisfactory consistency.^28^, ^29^ Descriptive statistics described clinical and demographic information. Relationships between demographic, clinical data, and children and young people's experiences of dental care were explored using parametric and nonparametric tests. The statistical significance was set at *p* < 0.05. Reporting followed the Consensus‐Based Checklist for Reporting of Survey Studies (CROSS).^30^

## 3 RESULTS

Seventy‐three surveys were completed; five were excluded due to duplications or being incomplete, resulting in a total of 68 valid surveys. Table 1 shows children and young people's demographic and clinical characteristics; the majority were male (55.9%), lived in major cities (77.9%), and most had a spastic CP motor type (69.1%) with half reporting unilateral spasticity (51.1%).

**TABLE 1:** Demographic information and clinical characteristics of children and young people with CP.

Demographic and clinical characteristics	Details	*n* = 68	%
Age (Mean ± SD)	11.3 ± 2.9 years		
Age range	7–17 years		
Age group	7–12 years	43	63.2
	13–17 years	24	35.3
	Not stated	1	1.5
Gender	Boys	38	55.9
	Girls	28	41.2
	Not stated	2	2.9
Country of birth	Australia	61	89.7
	Overseas	3	4.4
	Not stated	4	5.9
Remoteness	Major cities of Australia	53	77.9
	Inner regional Australia	11	16.2
	Not stated	4	5.9
SEIFA	Quintile 1	6	8.8
	Quintile 2	10	14.7
	Quintile 3	12	17.6
	Quintile 4	9	13.2
	Quintile 5	27	39.7
	Not stated	4	5.9
Type of CP	Ataxic	4	5.9
	Dyskinetic	5	7.4
	Spastic	47	69.1
	*Unilateral*	24	51.1
	*Bilateral*	23	48.9
	Unsure	12	17.6
GMFCS	Level I–II	31	48.4
	Level III–V	33	51.6
	Not stated	4	5.9
MACS	Level I–II	24	35.3
	Level III–V	40	58.8
	Not stated	4	5.9
VSS	Level I–II	33	48.5
	Level III–IV	31	45.6
	Not stated	4	5.9
CFCS	Level I–II	30	44.1
	Level III–V	34	50.0
	Not stated	4	5.9
Hearing	No impairment	58	85.3
	Some impairment	6	8.8
	Not stated	4	5.9
Epilepsy	Yes	34	53.1
Anti‐seizure medication	Yes	22	64.7

Abbreviations: CFCS, Communication Function Classification System^23^; GMFCS, Gross Motor Function Classification System^20^; MACS, Manual Ability Classification System^21^; SEIFA, Socio‐Economic Indexes For Areas^19^; VSS, Viking Speech Scale^22^.

Approximately half of our participants required assistive equipment for mobility (GMFCS III‐V) and 60% had difficulties handling objects (MACS levels III‐V). Just over half had epilepsy, with nearly 65% prescribed anti‐seizure medication (Table 1).

The COHIP‐SF 19 mean score for children and young people with CP was 51.9 (SD 12.4), with scores ranging from 16 to 76, indicating diverse experiences and moderate overall OHRQoL. The Oral Health Well‐Being domain had a mean score of 11.2 (SD 3.7) ranging from 4 to 20, reflecting a mix of positive and negative experiences. The Functional Well‐Being domain score of 10.6 (SD 4.2), ranging from 1 to 16, indicated notable challenges in daily oral health tasks. The Social‐emotional Well‐Being domain had a mean score of 30.0 ± 6.6, ranging from 8 to 40, suggesting a generally favorable impact on social and emotional well‐being, despite significant variability in experiences (Table 2).

**TABLE 2:** Distribution of scores by domain and for the total COHIP‐SF 19 scale.

COHIP‐SF 19 score	Mean ± SD	Range
**TOTAL** ^a^	51.9 ± 12.4	16–76
**OHWB domain (5 items)**	11.2 ± 3.7	4–20
**FWB domain (4 items)**	10.6 ± 4.2	1–16
**SEWB domain (10 items)**	30.0 ± 6.6	8–40

Abbreviations: COHIP, Child Oral Health Impact Profile; FWB, Functional Well‐Being; OHWB, Oral Health Well‐Being; SEWB, Social‐Emotional Well‐Being.

^a^ Total COHIP score can range from 0 to 76; higher scores reflect a more positive OHRQoL.

Cronbach's alpha was determined for each of the three domains: Oral Health Well‐being (*α* = 0.59), Functional Well‐being (*α* = 0.75), and Social‐emotional Well‐being (*α* = 0.84). The COHIP‐SF 19 scale had a high level of internal consistency, as determined by a Cronbach's alpha of 0.87.^24^, ^25^

Bivariate analysis showed that boys had significantly higher COHIP‐SF 19 total scores and mean Oral Health Well‐Being scores than girls. No significant differences in OHRQoL were found based on age, remoteness, CP motor type, GMFCS, MACS, hearing, epilepsy, or anti‐seizure medication (Table 3).

**TABLE 3:** Bivariate analyses showing the relationship between OHRQoL and demographic and clinical characteristics.

Demographic and clinical characteristics	*n*	COHIP‐SF 19 scale			Oral health Well‐Being domain			Functional Well‐Being domain			Social‐Emotional Well‐Being domain		
		Mean ± SD	Median	*p‐*value	Mean ± SD	Median	*p‐*value	Mean ± SD	Median	*p‐*value	Mean ± SD	Median	*p‐*value
**Age**	67	51.9 ± 12.4	53.0	0.208^d^			0.129^d^			0.704^d^			0.218^d^
7–12 years	43	50.7 ± 12.9	51.5	0.250^b^	10.5 ± 3.3	10.0	0.141^a^	10.6 ± 4.3	11.0	0.942^b^	29.1 ± 7.3	30.0	0.260^b^
13–17 years	24	53.7 ± 11.0	56.5		11.9 ± 4.0	12.0		10.6 ± 4.1	11.0		31.1 ± 5.0	32.0	
**Gender**													
Boys	38	54.1 ± 12.4	55.5	**0.046** ^b^	12.1 ± 3.7	12.0	**0.008** ^a^	11.3 ± 4.1	12.0	0.089^b^	30.6 ± 6.9	32.0	0.193^b^
Girls	28	48.5 ± 11.0	49.0		9.7 ± 3.1	10.0		9.7 ± 4.2	9.5		29.0 ± 6.1	30.0	
**Remoteness**													
Major cities of Australia	53	52.2 ± 12.9	54.0	0.551^a^	11.4 ± 3.7	12.0	0.071^a^	10.9 ± 4.3	11.0	0.221^b^	29.8 ± 6.8	31.0	0.830^b^
Inner Regional Australia	11	49.8 ± 7.8	51.0		9.6 ± 3.2	10.0		9.5 ± 4.0	11.0		30.6 ± 4.6	30.0	
**SEIFA**													
Quintile 1	6	51.5 ± 11.9	53.0	0.157e	11.3 ± 4.7	10.0	0.645f	9.6 ± 5.7	12.5	0.120c	30.5 ± 5.8	32.0	0.416c
Quintile 2	10	51.0 ± 11.1	49.5		10.5 ± 4.1	11.0		11.0 ± 4.0	12.0		29.5 ± 8.2	29.5	
Quintile 3	12	45.5 ± 14.7	46.5		10.0 ± 3.6	9.5		8.5 ± 4.4	7.0		26.9 ± 8.2	30.0	
Quintile 4	9	51.3 ± 11.4	56.0		10.7 ± 2.9	10.0		9.7 ± 2.8	10.0		30.7 ± 8.8	32.0	
Quintile 5	27	55.1 ± 11.4	59.0		11.9 ± 3.6	12.0		12.0 ± 4.1	13.0		31.1 ± 5.2	31.0	
**CP Type**													
Ataxic	4	57.2 ± 7.5	60.5	0.668^c^	13.5 ± 3.6	14.0	0.595^c^	11.0 ± 3.3	9.5	0.747^c^	32.7 ± 4.3	33.5	0.751^c^
Dyskinetic	5	53.4 ± 11.0	57.0		10.0 ± 3.2	12.0		11.4 ± 4.1	13.0		32.0 ± 4.1	31.0	
Spastic	47	52.0 ± 11.9	53.0		11.1 ± 3.8	11.0		10.9 ± 4.1	12.0		29.8 ± 6.3	30.0	
*Unilateral*	6	54.6 ± 11.8	54.5		12.0 ± 3.1	12.0		11.3 ± 4.2	11.0		31.3 ± 5.9	31.0	
*Bilateral*	41	51.6 ± 12.0	53.0		11.0 ± 3.9	11.0		10.9 ± 4.1	12.0		29.6 ± 6.4	30.0	
Unsure	12	48.9 ± 16.3	53.0		11.0 ± 4.0	10.0		9.1 ± 5.2	8.0		28.7 ± 9.3	31.5	
**GMFCS**													
Level I‐II	31	48.4 ± 15.7	45.0	0.124^b^	11.9 ± 3.4	12.0	0.113^a^	11.7 ± 3.6	12.0	0.094^b^	30.3 ± 6.7	31.0	0.558^b^
Level III‐V	33	55.4 ± 11.0	58.0		10.4 ± 3.9	10.0		9.7 ± 4.6	11.0		29.6 ± 6.3	30.0	
**MACS**													
Level I‐II	24	54.3 ± 12.0	54.5	0.203^a^	12.2 ± 3.0	12.0	0.075^a^	11.7 ± 3.5	12.5	0.179^b^	30.4 ± 6.9	31.0	0.682^b^
Level III‐V	40	50.3 ± 12.2	52.0		10.5 ± 3.9	10.0		10.0 ± 4.5	11.0		29.7 ± 6.3	30.5	
**VSS**													
Level I‐II	33	54.2 ± 11.9	55.0	0.134^a^	11.8 ± 3.3	12	0.102^a^	12.2 ± 3.4	13.0	**0.005** ^b^	30.2 ± 6.6	31.0	0.893^b^
Level II‐IV	31	49.2 ± 12.1	51.0		10.4 ± 4.0	10		9.1 ± 4.5	9.0		29.7 ± 6.4	31.0	
**CFCS**													
Level I‐II	30	55.9 ± 12.0	58.5	**0.010** ^a^	12.4 ± 3.3	12.0	**0.010** ^b^	12.5 ± 3.2	13.0	**0.002** ^b^	31.0 ± 6.8	31.0	0.202^b^
Level III‐V	34	48.2 ± 11.3	51		10.0 ± 3.7	10.0		9.0 ± 4.4	9.0		29.0 ± 6.1	30.0	
**Hearing**													
No impairment	58	52.3 ± 12.2	53.5	0.280^a^	11.4 ± 3.7	12.0	0.087^a^	10.7 ± 4.2	11.0	0.762^b^	30.2 ± 6.5	31.0	0.360^b^
Some impairment	6	46.6 ± 11.6	46.0		8.6 ± 1.9	8.0		10.0 ± 5.3	11.5		28.0 ± 6.2	30.0	
**Epilepsy**													
Yes	34	46.6 ± 11.6	46.0	0.742^b^	10.7 ± 3.3	8.0	0.394^a^	10.0 ± 5.3	11.5	0.321^b^	28.0 ± 6.2	29.5	0.366^b^
No	30	52.3 ± 12.2	53.5		11.4 ± 3.7	12.0		10.7 ± 4.2	11.0		30.2 ± 6.5	31.0	
**Anti‐seizure medication**													
Yes	22	52.2 ± 9.5	54.5	0.669^a^	10.8 ± 3.4	11.0	0.902^a^	9.9 ± 4.6	10.5	0.817^b^	31.4 ± 4.8	32.0	0.511^b^
No	12	50.5 ± 13.6	52.0		10.6 ± 3.3	11.0		10.8 ± 5.2	10.5		29.7 ± 6.5	31.5	

*Note*: Bold values are statisitical significant.

Abbreviations: CFCS, communication function classification system^23^; GMFCS, gross motor function classification system^20^; MACS, manual ability classification system^21^; OHRQoL, oral health‐related quality of life; VSS, Viking Speech Scale^22^.

^a^ Independent samples t‐test.

^b^ Mann–Whitney *U*‐test.

^c^ Kruskal–Wallis *H*‐test.

^d^ Linear regression.

^e^ Welch's One‐way ANOVA.^31^

^f^ One‐way ANOVA.^31^

Participants with CFCS levels I‐II had higher OHRQoL scores across all domains compared to those with CFCS levels III‐V, suggesting that greater communication skills were linked to improved oral health well‐being, daily functioning, and social‐emotional interactions.

More than half (55.9%) of caregivers reported that managing their child's daily oral health routine was “a bit of a challenge.” Three‐quarters of caregivers felt that CP impacted their child's ability to participate in dental exams, with nearly 40% reporting their child had received dental treatment in a hospital setting. Only 4.6% of participants relied exclusively on School Dental Services (SDS) with 30.8% reporting SDS was unable to provide care for their child, and 53.8% indicating the need to make alternative arrangements and/or not using SDS (Table 4).

**TABLE 4:** Experiences of dental care and services.

Experiences of dental care and services	*n*	%
**Is your daily routine of teeth cleaning**:		
Fairly straightforward	12	17.6
A bit of a challenge	38	55.9
Extremely difficult	17	25.0
**Do you have a regular dentist?**		
My whole family uses the same dentist.	24	35.3
I have my own dentist or more than one dentist.	22	32.4
Different family members in my family have different dentists.	8	11.8
I do not have a dentist.	13	19.1
**Does CP affect your capacity to participate in dental examinations and treatment?** Yes*	51	75.0
**If you have previously accessed dental treatment, for example, exam, filling, cap/crown**. **Please select all that apply**.		
Simple dental check‐up clean, no special requirements needed.	44	64.7
Special arrangements were needed to attend the local practice	16	23.5
Some sedation was needed at the general dentist.	5	7.4
Dental treatment needed to be performed at a day surgery/hospital.	27	39.7
Never accessed dental treatment.	2	2.9
**Have you ever had a dental professional (including Dental Hygienist, Dental Therapist, Oral Health Therapist or Dentist) come to your home for examination or treatment?** Yes*	2	2.9
**Have you had to find urgent dental care due to pain or another problem(s)?** Yes*	17	25.0
**Have you required complex dental surgery? On what basis has the complex surgery been provided?**		
Emergency	–	
Planned	12	17.9
Emergency and planned	6	8.9
Not applicable	43	64.1
**If you needed complex dental surgery, how were the arrangements made?**		
I made the arrangements	9	13.4
I was given assistance by another medical/dental practitioner.	9	13.4
**SDS**		
We rely upon SDS for all dental care	3	4.6
The SDS has been flexible and able to assist my child	7	10.8
The SDS is not able to provide care for my child	20	30.8
Not applicable	35	53.8

*Note*: The table includes three yes/no questions pertaining to participation in dental examinations, home visits, and urgent dental care.

Bold values are statisitical significant.

Abbreviations: CP, Cerebral Palsy; SDS, School Dental Services.

* Only yes responses have been included in the table.

Bivariate analysis also showed that factors such as the daily oral health routine (*p* = 0.031) the impact of CP on the ability to participate in dental examinations (*p* = 0.032), the need for special arrangements to attend dental practices (*p* = 0.036) and seeking urgent dental care due to pain or other problem(s) (*p* = 0.007) significantly affect OHRQoL. These findings indicate that these factors are critical determinants of oral health outcomes and should be prioritized in the care and management of children and young people with CP (Table 5).

**TABLE 5:** Bivariate analyses showing the relationship between OHRQoL and children's and young people experiences of dental care and services.

Experiences of dental care and services	*N*	COHIP‐SF 19 scale			OHWB domain			FWB domain			SEWB domain		
		Mean ± SD	Median	*p*‐value	Mean ± SD	Median	*p*‐value	Mean ± SD	Median	*p*‐value	Mean ± SD	Median	*p*‐value
**Oral health daily routine**													
Fairly straightforward	12	58.3 ± 13.8	63.5	**0.031** ^c^	12.4 ± 3.8	12.5	**0.019** ^d^	13.0 ± 4.2	15.5	**0.013** ^c^	32.8 ± 6.7	35.5	0.234^c^
A bit of a challenge but manageable	38	52.8 ± 10.6	54.0		11.7 ± 3.8	11.5		10.8 ± 3.6	11.5		30.1 ± 5.5	31.0	
Extremely difficult, sometimes impossible	17	45.5 ± 13.1	48.0		9.1 ± 3.0	10.0		8.5 ± 4.8	7.0		27.8 ± 8.5	30.0	
**Regular dentist**													
Have a regular dentist or more than one dentist	24	50.2 ± 12.7	50.5	0.626^c^	10.3 ± 3.9	10.0	0.483^d^	10.0 ± 4.1	9.5	0.370^c^	29.7 ± 6.8	30.0	0.717^c^
The whole family uses the same dentist	22	54.5 ± 11.8	55.5		11.9 ± 4.1	12.0		12.0 ± 3.6	13.0		30.5 ± 6.1	31.5	
Different family members have different dentists	8	53.6 ± 13.6	53.5		12.2 ± 3.6	13.0		9.1 ± 5.6	9.0		32.2 ± 6.5	32.0	
No regular dentist	13	50.0 ± 13.0	53.0		11.0 ± 2.9	11.0		10.6 ± 4.5	12.0		28.3 ± 7.8	31.0	
**CP affects capacity to participate in dental exam and treatment**													
Yes	51	50.1 ± 12.5	52.0	**0.032** ^a^	10.9 ± 3.8	11.0	0.289^a^	10.1 ± 4.4	11.0	**0.046** ^b^	29.1 ± 6.9	30.0	0.053^b^
No	16	57.8 ± 10.9	57.5		12.1 ± 3.4	12.0		12.6 ± 3.0	13.0		33.0 ± 5.2	33.5	
**No special requirements for simple dental check‐ups**													
Yes	44	54.6 ± 11.2	47.0	**0.011** ^a^	12.3 ± 3.6	12.0	** < 0.001** ^b^	11.2 ± 3.9	12.0	0.170^b^	31.0 ± 6.0	31.0	0.139^b^
No	24	46.7 ± 13.0	56.0		9.1 ± 3.2	9.0		9.6 ± 4.7	9.5		28.0 ± 7.5	30.0	
**Special arrangements were needed to attend the local practice**													
Yes	16	46.0 ± 12.8	42.5	**0.036** ^b^	9.8 ± 3.1	9.5	0.082^b^	8.3 ± 4.8	7.5	**0.024** ^b^	27.8 ± 7.7	29.5	0.155^b^
No	52	53.7 ± 11.8	54.5		11.6 ± 3.8	12.0		11.4 ± 3.8	12.0		30.6 ± 6.2	31.0	
**Some sedation was needed at the general dentist**													
Yes	5	42.8 ± 13.1	42.0	0.089^a^	9.6 ± 1.9	9.0	0.323^b^	8.0 ± 4.5	8.0	0.149^b^	25.2 ± 11.1	25.0	0.248^b^
No	63	52.6 ± 12.2	54.0		11.3 ± 3.8	11.0		10.9 ± 4.2	12.0		30.3 ± 6.1	31.0	
**Dental treatment needed to be performed at a day surgery hospital**													
Yes	27	48.9 ± 13.5	53.0	0.115^a^	10.3 ± 3.8	10.0	0.109^a^	9.5 ± 4.7	9.0	0.095^b^	29.1 ± 7.7	31.0	0.792^b^
No	41	53.8 ± 11.4	53.0		11.8 ± 3.6	12.0		11.4 ± 3.8	12.0		30.5 ± 5.8	31.0	
**Have you had to find urgent dental care due to pain or another problem(s)?**													
Yes	17	45.4 ± 11.3	42.0	**0.007** ^b^	9.6 ± 3.3	9.0	**0.030** ^b^	7.7 ± 4.5	7.0	**0.002** ^b^	28.0 ± 5.9	27.0	**0.024** ^b^
No	50	54.2 ± 12.2	56.0		11.7 ± 3.8	12.0		11.7 ± 3.7	13.0		30.7 ± 6.9	32.0	
**Complex surgery**													
Planned	13	52.0 ± 14.0	59.0	0.235^a^	11.0 ± 4.2	12.0	0.086^e^	11.4 ± 4.2	13.0	**0.023** ^a^	29.5 ± 7.8	31.0	0.529^e^
Emergency and planned	7	44.5 ± 10.7	44.0		8.7 ± 1.3	9.0		6.7 ± 3.6	9.0		29.1 ± 7.5	25.0	
**SDS**													
We rely upon the SDS for all dental care	3	64.3 ± 9.0	63.0	0.137^d^	14.3 ± 4.0	15.0	0.270^d^	14.6 ± 1.1	14.0	0.144^c^	35.3 ± 4.1	34.0	0.401^d^
The SDS has been flexible and able to assist my child	7	45.8 ± 18.8	42.0		11.0 ± 5.3	10.0		8.0 ± 5.9	4.0		26.8 ± 9.4	29.0	
The SDS is not able to provide care for my child	20	50.9 ± 10.8	53.0		10.3 ± 3.2	11.0		10.2 ± 4.5	11.0		30.3 ± 6.4	31.5	

*Note*: Bold values are statisitical significant.

Abbreviation: CP, Cerebral Palsy; FWB, Functional Well‐Being; OHRQoL, Oral Health‐Related Quality of Life; SDS, School Dental Services.

^a^ Independent samples *t*‐test.

^b^ Mann–Whitney *U*‐test.

^c^ Kruskal–Wallis *H*‐test.

^d^ One way ANOVA.

^e^ Welch *t*‐test.

## 4 DISCUSSION

This is the first study to explore OHRQoL and dental care experiences through caregiver reports for Australian children and young people with CP, using the COHIP‐SF 19, where higher scores indicate a more positive OHRQoL. The COHIP‐SF 19 revealed an average score of 51.9%. ± 12.4, reflecting a moderate OHRQoL. Over half of the caregivers reported that daily oral health routines were “a bit of a challenge”. Most caregivers noted that CP complicates their child's participation in dental examinations, making access to dental care more complex.

CP is a heterogenous condition, characterized by a wide range of motor limitations and impairments. Here, more than half of our participants had significant functional limitations, including requiring the use of assistive mobility equipment (GMFCS III‐V), having moderate to severe manual limitations (MACS III‐V), and communication difficulties (CFCS III‐V). These functional impairments were more severe than would have been expected from the general CP population.^32^

Research has emphasized that individual factors, such as grip force dynamics, spasticity, motivation, and oromotor challenges play a crucial role in oral hygiene.^33^ Interestingly, this study found slightly higher COHIP‐SF 19 scores for participants with GMFCS levels III‐V (55.4 ± 11.0) compared to those with GMFCS levels I‐II (48.4 ± 15.7). This discrepancy suggests that while higher functional impairments might generally correlate with lower OHRQoL, our study did not find a significant link between the severity of motor limitations and OHRQoL scores. The authors hypothesize that caregivers’ efforts to support their children and young people's oral hygiene may have improved the perceived OHRQoL, potentially explaining the observed variation. VSS findings indicate that children with milder speech impairments (Levels I‐II) report better functional well‐being and fewer challenges with activities like eating, sleeping, and oral hygiene, leading to higher COHIP‐SF 19 scores and overall OHRQoL compared to those with Levels III‐IV.

Participants with CFCS level I‐II reported significantly better OHRQoL across all domains, supporting the notion that functional communication skills are associated with better oral health well‐being, and overall quality of life.^34^, ^35^

Some gender‐based variations were observed, with boys reporting higher COHIP‐SF 19 scores overall in the oral health well‐being domain. These findings are consistent with some previous research but contrast with other studies where girls had higher scores.^36^, ^37^ It is important to note that both studies did not involve children with CP.^36^, ^37^ Specific COHIP‐SF 19 items, such as “had pain in their teeth/toothache” and “had bleeding gums,” revealed subtle gender differences, favoring higher scores for boys. The reasons for these discrepancies remain unclear but may be associated with a combination of gender and age‐related factors. For example, younger children may report oral health issues differently due to their experiences and ability to articulate problems. Hormonal changes during puberty can affect gingival health at different developmental stages.^38^ Additionally, different attitudes toward oral health between genders may lead to boys underreporting issues related to pain and discomfort.^37^, ^38^

The average COHIP scores for participants with CP in our study were lower than those reported for children without CP.^39^ For example, previous research found an average COHIP score of 60.7% in children without CP, with notably higher scores in Oral Health Well‐Being (e.g., dental pain, crooked teeth, gingivitis) and Functional Well‐Being domains (e.g., difficulty with eating, saying certain words or keeping teeth clean).^37^ This emphasizes the unique challenges faced by children and young people with CP in maintaining oral health. Interestingly, our findings did reveal that children and young people with CP had similar scores to those in previous research in the Socio‐Emotional Well‐Being category (e.g., unhappy or sad because of teeth, worried about what people think).^37^ This may suggest that although CP impacts functional and physical aspects of oral health, the socio‐emotional aspects of OHRQoL are comparable to those of children without CP.

The data revealed that just over half of our participants had epilepsy, with nearly 65% taking medication. It was anticipated by the authors that participants with CP and epilepsy might have significantly lower COHIP‐SF 19 scores, due to associated oral health issues for example gingivitis, and decayed and missing teeth.^40^ Although the differences were not statistically significant, participants with epilepsy who were on medication had an average COHIP‐SF 19 score of 54.5 (± 9.5), slightly higher than those not on medication, who had an average score of 52.0 (± 13.6). Further research and more extensive studies are needed to explore the potential impact of medications for epilepsy and their impact on OHRQoL in children and young people with CP.

Approximately 20% of participants did not have a regular dentist, which may lead to delayed treatment and missed preventive opportunities, negatively affecting OHRQoL. This finding is consistent with other research which reported a quarter of their participants with disabilities also lacked a regular dentist.^26^ Additionally 30% of caregivers reported that the school‐based dental services were not able to provide care for their child, and another 50% said this option was not applicable. These challenges might be due to the availability of services, workforce shortages, and access issues, exacerbated by mobility and communication limitations.^41^ Australia's National Oral Health Plan 2015–2024 reports a shortage of dental professionals with the skill set to provide oral health services for people with disability.^15^

According to the National Child Oral Health Study 2012–2014, approximately 80% of children and young people receive preventive care. However, our study reports much lower figures for participants with CP at approximately 65%.^38^ Additionally, 17.9% of participants in our study with CP required complex dental surgery, and 25% needed urgent dental care due to pain or other issues, suggesting a demand for specialized dental services.

The 2023 Senate Select Committee report identified that pathways for people with disabilities are insufficient, leading to the overuse of general anesthesia and extensive waitlists, which exacerbate oral health issues.^15^ The committee recommended providing care through general dental practices with reasonable adjustments, rather than relying solely on specialist care. Our findings support this recommendation, emphasizing the need for more inclusive dental care environments to shorten waitlists, and improve OHRQoL for children and young people with CP.

### 4.1 Strengths and limitations

To our knowledge, this is the first study in Australia to explore oral health impact among children and young people with CP using the COHIP‐SF 19.^26^

While the cross‐sectional design and small sample size limit causal interference and may introduce selection bias, this study provides valuable baseline insights into the OHRQoL in children with CP. Whilst previous research found strong correlations between child and proxy reports, other studies have reported that caregivers may overestimate a child's OHRQoL.^42^, ^43^, ^44^ The subjective nature of OHRQoL assessments may result in variability in responses, as they are influenced by individual perceptions and experiences of quality of life. Future studies should aim to collect direct reports from children and young people to gain a more comprehensive understanding of OHRQoL in this population.

This study included children and young people with more severe functional limitations than the general CP population and were predominantly from major cities and socioeconomically advantaged backgrounds, suggesting some level of selection bias. Consequently, our findings may not fully reflect the experiences of all children and young people with CP, particularly those from disadvantaged or remote areas. Given the link between socioeconomic circumstances and access to dental care, future research should seek to include a diverse cross‐section of the population to ensure the identification of barriers and enablers to accessing care.^44^

Additionally, it is not known whether participants had received interventions that may have impacted their OHRQoL.

Some of the enablers and barriers to dental care will be explored in a separate qualitative paper that is being conducted by this author group.

### 4.2 Recommendations

In line with best practice, multidisciplinary collaboration, supported by integrated technologies like teledentistry, should be utilized within comprehensive, accessible, and patient‐centered care to address individual challenges and reduce inequalities to improve OHRQoL for children with CP.^6^, ^45^

## 5 CONCLUSION

Our study assessed the OHRQoL in children and young people with CP in Australia using the COHIP‐SF 19 scale which was validated as a reliable tool for assessing OHRQoL in this population. The results indicated that children and young people with CP had moderate OHRQoL. Factors that significantly impacted OHRQoL included gender, communication limitations, oral health daily routine, participation in dental exams and treatment, special arrangements to attend the practice and the need for urgent dental care due to pain or other problem(s). No significant correlations were found between GMFCS and COHIP‐SF 19 scores, highlighting the complexity of OHRQoL in this population and suggesting that factors beyond clinical characteristics significantly influence perceived OHRQoL.

## CONFLICT OF INTEREST STATEMENT

The authors declare no conflict of interest.

## INSTITUTIONAL REVIEW BOARD STATEMENT

The study was conducted in accordance with the University of Sydney Human Ethics and approved by the Victorian Cerebral Palsy Register Governance and Queensland Cerebral Palsy Register Steering Committee.

## INFORMED CONSENT STATEMENT

Informed consent was obtained from all subjects involved in the study.

## Data Availability

The data presented in this study are available on request from the corresponding author. The data are not publicly available due to ethical restrictions.
